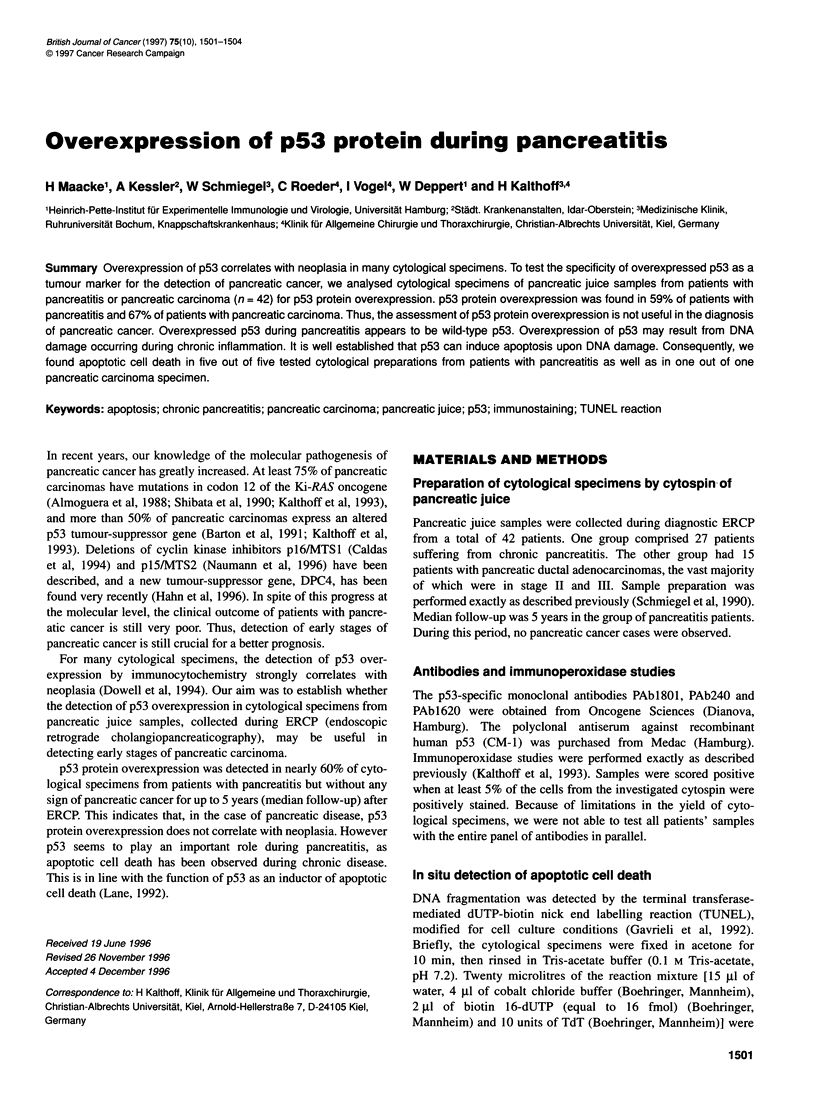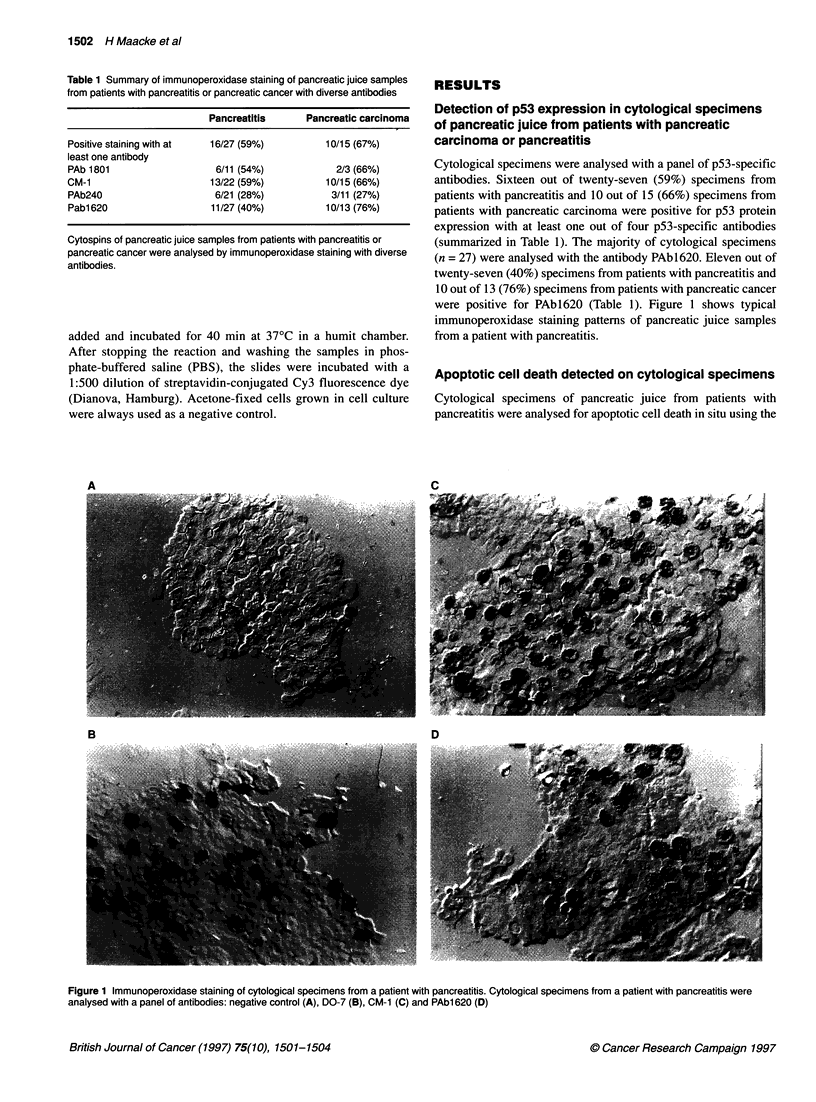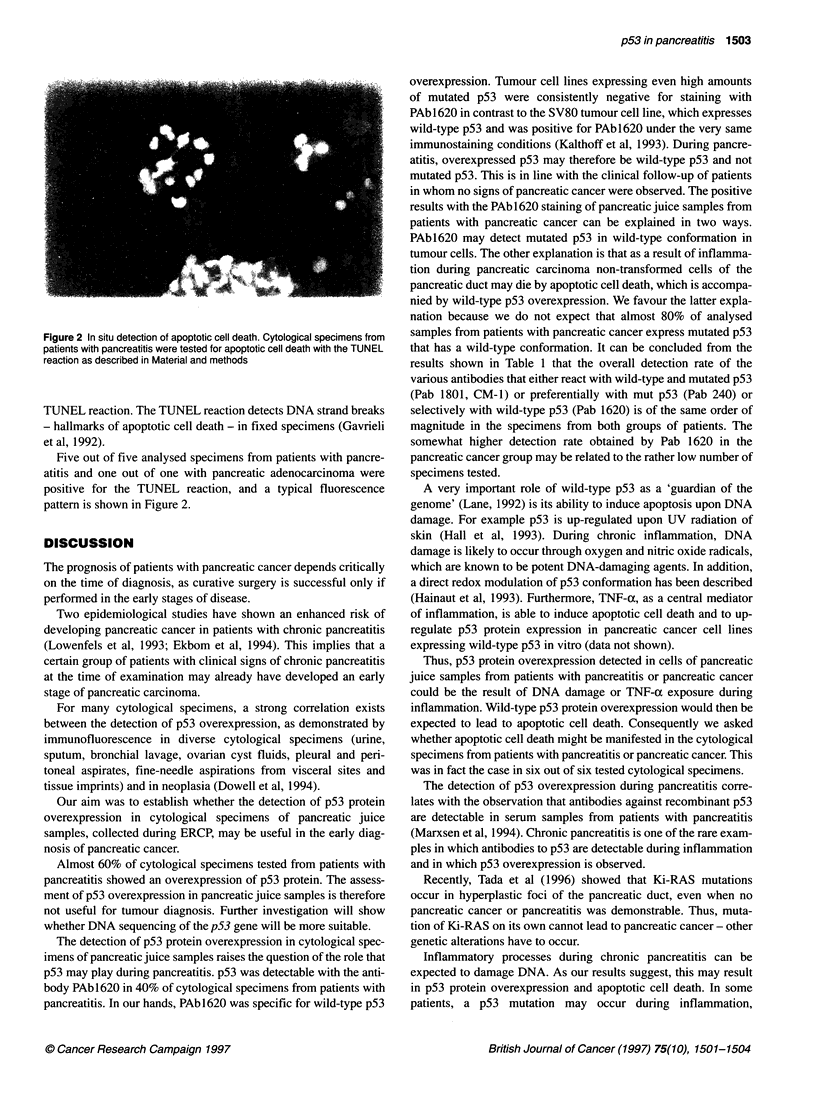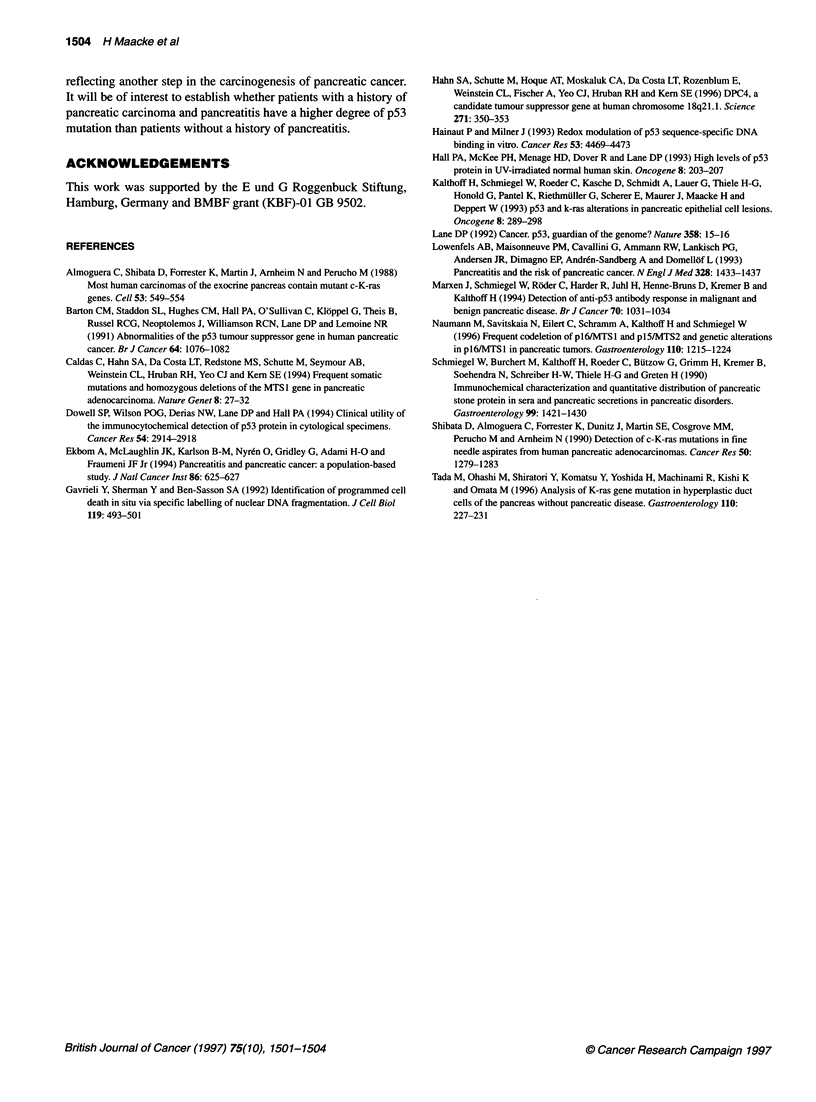# Overexpression of p53 protein during pancreatitis.

**DOI:** 10.1038/bjc.1997.256

**Published:** 1997

**Authors:** H. Maacke, A. Kessler, W. Schmiegel, C. Roeder, I. Vogel, W. Deppert, H. Kalthoff

**Affiliations:** Heinrich-Pette-Institut fÃ¼r Experimentelle Immunologie und Virologie, UniversitÃ¤t Hamburg, Germany.

## Abstract

**Images:**


*British Journal of Cancer* (1997) **75**(10), 1501–1504
© 1997 Cancer Research Campaign

### Overexpression of p53 protein during pancreatitis

**H Maacke[1], A Kessler[2], W Schmiegel[3], C Roeder[4], I Vogel[4], W Deppert[1] and H Kalthoff[3,4]**

[1]Heinrich-Pette-Institut für Experimentelle Immunologie und Virologie, Universität Hamburg; [2]Städt. Krankenanstalten, Idar-Oberstein; [3]Medizinische Klinik, Ruhruniversität Bochum, Knappschaftskrankenhaus; [4]Klinik für Allgemeine Chirurgie und Thoraxchirurgie, Christian-Albrechts Universität, Kiel, Germany

**Summary** Overexpression of p53 correlates with neoplasia in many cytological specimens. To test the specificity of overexpressed p53 as a tumour marker for the detection of pancreatic cancer, we analysed cytological specimens of pancreatic juice samples from patients with pancreatitis or pancreatic carcinoma ($n = 42$) for p53 protein overexpression. p53 protein overexpression was found in 59% of patients with pancreatitis and 67% of patients with pancreatic carcinoma. Thus, the assessment of p53 protein overexpression is not useful in the diagnosis of pancreatic cancer. Overexpressed p53 during pancreatitis appears to be wild-type p53. Overexpression of p53 may result from DNA damage occurring during chronic inflammation. It is well established that p53 can induce apoptosis upon DNA damage. Consequently, we found apoptotic cell death in five out of five tested cytological preparations from patients with pancreatitis as well as in one out of one pancreatic carcinoma specimen.

**Keywords:** apoptosis; chronic pancreatitis; pancreatic carcinoma; pancreatic juice; p53; immunostaining; TUNEL reaction

In recent years, our knowledge of the molecular pathogenesis of pancreatic cancer has greatly increased. At least 75% of pancreatic carcinomas have mutations in codon 12 of the Ki-*RAS* oncogene (Almoguera et al, 1988; Shibata et al, 1990; Kalthoff et al, 1993), and more than 50% of pancreatic carcinomas express an altered p53 tumour-suppressor gene (Barton et al, 1991; Kalthoff et al, 1993). Deletions of cyclin kinase inhibitors p16/MTS1 (Caldas et al, 1994) and p15/MTS2 (Naumann et al, 1996) have been described, and a new tumour-suppressor gene, DPC4, has been found very recently (Hahn et al, 1996). In spite of this progress at the molecular level, the clinical outcome of patients with pancreatic cancer is still very poor. Thus, detection of early stages of pancreatic cancer is still crucial for a better prognosis.

For many cytological specimens, the detection of p53 overexpression by immunocytochemistry strongly correlates with neoplasia (Dowell et al, 1994). Our aim was to establish whether the detection of p53 overexpression in cytological specimens from pancreatic juice samples, collected during ERCP (endoscopic retrograde cholangiopancreaticography), may be useful in detecting early stages of pancreatic carcinoma.

p53 protein overexpression was detected in nearly 60% of cytological specimens from patients with pancreatitis but without any sign of pancreatic cancer for up to 5 years (median follow-up) after ERCP. This indicates that, in the case of pancreatic disease, p53 protein overexpression does not correlate with neoplasia. However p53 seems to play an important role during pancreatitis, as apoptotic cell death has been observed during chronic disease. This is in line with the function of p53 as an inductor of apoptotic cell death (Lane, 1992).

*Received 19 June 1996*
*Revised 26 November 1996*
*Accepted 4 December 1996*

*Correspondence to:* H Kalthoff, Klinik für Allgemeine und Thoraxchirurgie, Christian-Albrechts Universität, Kiel, Arnold-Hellerstraße 7, D-24105 Kiel, Germany

### MATERIALS AND METHODS

### Preparation of cytological specimens by cytospin of pancreatic juice

Pancreatic juice samples were collected during diagnostic ERCP from a total of 42 patients. One group comprised 27 patients suffering from chronic pancreatitis. The other group had 15 patients with pancreatic ductal adenocarcinomas, the vast majority of which were in stage II and III. Sample preparation was performed exactly as described previously (Schmiegel et al, 1990). Median follow-up was 5 years in the group of pancreatitis patients. During this period, no pancreatic cancer cases were observed.

### Antibodies and immunoperoxidase studies

The p53-specific monoclonal antibodies PAb1801, PAb240 and PAb1620 were obtained from Oncogene Sciences (Dianova, Hamburg). The polyclonal antiserum against recombinant human p53 (CM-1) was purchased from Medac (Hamburg). Immunoperoxidase studies were performed exactly as described previously (Kalthoff et al, 1993). Samples were scored positive when at least 5% of the cells from the investigated cytospin were positively stained. Because of limitations in the yield of cytological specimens, we were not able to test all patients' samples with the entire panel of antibodies in parallel.

### In situ detection of apoptotic cell death

DNA fragmentation was detected by the terminal transferase-mediated dUTP-biotin nick end labelling reaction (TUNEL), modified for cell culture conditions (Gavrieli et al, 1992). Briefly, the cytological specimens were fixed in acetone for 10 min, then rinsed in Tris-acetate buffer (0.1 M Tris-acetate, pH 7.2). Twenty microlitres of the reaction mixture [15 μl of water, 4 μl of cobalt chloride buffer (Boehringer, Mannheim), 2 μl of biotin 16-dUTP (equal to 16 fmol) (Boehringer, Mannheim) and 10 units of TdT (Boehringer, Mannheim)] were

**Table 1** Summary of immunoperoxidase staining of pancreatic juice samples from patients with pancreatitis or pancreatic cancer with diverse antibodies

| | Pancreatitis | Pancreatic carcinoma |
|---|---|---|
| Positive staining with at least one antibody | 16/27 (59%) | 10/15 (67%) |
| PAb 1801 | 6/11 (54%) | 2/3 (66%) |
| CM-1 | 13/22 (59%) | 10/15 (66%) |
| PAb240 | 6/21 (28%) | 3/11 (27%) |
| Pab1620 | 11/27 (40%) | 10/13 (76%) |

Cytospins of pancreatic juice samples from patients with pancreatitis or pancreatic cancer were analysed by immunoperoxidase staining with diverse antibodies.

added and incubated for 40 min at 37°C in a humit chamber. After stopping the reaction and washing the samples in phosphate-buffered saline (PBS), the slides were incubated with a 1:500 dilution of streptavidin-conjugated Cy3 fluorescence dye (Dianova, Hamburg). Acetone-fixed cells grown in cell culture were always used as a negative control.

### RESULTS

### Detection of p53 expression in cytological specimens of pancreatic juice from patients with pancreatic carcinoma or pancreatitis

Cytological specimens were analysed with a panel of p53-specific antibodies. Sixteen out of twenty-seven (59%) specimens from patients with pancreatitis and 10 out of 15 (66%) specimens from patients with pancreatic carcinoma were positive for p53 protein expression with at least one out of four p53-specific antibodies (summarized in Table 1). The majority of cytological specimens ($n$ = 27) were analysed with the antibody PAb1620. Eleven out of twenty-seven (40%) specimens from patients with pancreatitis and 10 out of 13 (76%) specimens from patients with pancreatic cancer were positive for PAb1620 (Table 1). Figure 1 shows typical immunoperoxidase staining patterns of pancreatic juice samples from a patient with pancreatitis.

### Apoptotic cell death detected on cytological specimens

Cytological specimens of pancreatic juice from patients with pancreatitis were analysed for apoptotic cell death in situ using the

**Figure 1** Immunoperoxidase staining of cytological specimens from a patient with pancreatitis. Cytological specimens from a patient with pancreatitis were analysed with a panel of antibodies: negative control (**A**), DO-7 (**B**), CM-1 (**C**) and PAb1620 (**D**)

 © Cancer Research Campaign 1997

**Figure 2** In situ detection of apoptotic cell death. Cytological specimens from patients with pancreatitis were tested for apoptotic cell death with the TUNEL reaction as described in Material and methods

TUNEL reaction. The TUNEL reaction detects DNA strand breaks – hallmarks of apoptotic cell death – in fixed specimens (Gavrieli et al, 1992).

Five out of five analysed specimens from patients with pancreatitis and one out of one with pancreatic adenocarcinoma were positive for the TUNEL reaction, and a typical fluorescence pattern is shown in Figure 2.

### DISCUSSION

The prognosis of patients with pancreatic cancer depends critically on the time of diagnosis, as curative surgery is successful only if performed in the early stages of disease.

Two epidemiological studies have shown an enhanced risk of developing pancreatic cancer in patients with chronic pancreatitis (Lowenfels et al, 1993; Ekbom et al, 1994). This implies that a certain group of patients with clinical signs of chronic pancreatitis at the time of examination may already have developed an early stage of pancreatic carcinoma.

For many cytological specimens, a strong correlation exists between the detection of p53 overexpression, as demonstrated by immunofluorescence in diverse cytological specimens (urine, sputum, bronchial lavage, ovarian cyst fluids, pleural and peritoneal aspirates, fine-needle aspirations from visceral sites and tissue imprints) and in neoplasia (Dowell et al, 1994).

Our aim was to establish whether the detection of p53 protein overexpression in cytological specimens of pancreatic juice samples, collected during ERCP, may be useful in the early diagnosis of pancreatic cancer.

Almost 60% of cytological specimens tested from patients with pancreatitis showed an overexpression of p53 protein. The assessment of p53 overexpression in pancreatic juice samples is therefore not useful for tumour diagnosis. Further investigation will show whether DNA sequencing of the *p53* gene will be more suitable.

The detection of p53 protein overexpression in cytological specimens of pancreatic juice samples raises the question of the role that p53 may play during pancreatitis. p53 was detectable with the antibody PAb1620 in 40% of cytological specimens from patients with pancreatitis. In our hands, PAb1620 was specific for wild-type p53 overexpression. Tumour cell lines expressing even high amounts of mutated p53 were consistently negative for staining with PAb1620 in contrast to the SV80 tumour cell line, which expresses wild-type p53 and was positive for PAb1620 under the very same immunostaining conditions (Kalthoff et al, 1993). During pancreatitis, overexpressed p53 may therefore be wild-type p53 and not mutated p53. This is in line with the clinical follow-up of patients in whom no signs of pancreatic cancer were observed. The positive results with the PAb1620 staining of pancreatic juice samples from patients with pancreatic cancer can be explained in two ways. PAb1620 may detect mutated p53 in wild-type conformation in tumour cells. The other explanation is that as a result of inflammation during pancreatic carcinoma non-transformed cells of the pancreatic duct may die by apoptotic cell death, which is accompanied by wild-type p53 overexpression. We favour the latter explanation because we do not expect that almost 80% of analysed samples from patients with pancreatic cancer express mutated p53 that has a wild-type conformation. It can be concluded from the results shown in Table 1 that the overall detection rate of the various antibodies that either react with wild-type and mutated p53 (Pab 1801, CM-1) or preferentially with mut p53 (Pab 240) or selectively with wild-type p53 (Pab 1620) is of the same order of magnitude in the specimens from both groups of patients. The somewhat higher detection rate obtained by Pab 1620 in the pancreatic cancer group may be related to the rather low number of specimens tested.

A very important role of wild-type p53 as a 'guardian of the genome' (Lane, 1992) is its ability to induce apoptosis upon DNA damage. For example p53 is up-regulated upon UV radiation of skin (Hall et al, 1993). During chronic inflammation, DNA damage is likely to occur through oxygen and nitric oxide radicals, which are known to be potent DNA-damaging agents. In addition, a direct redox modulation of p53 conformation has been described (Hainaut et al, 1993). Furthermore, TNF-α, as a central mediator of inflammation, is able to induce apoptotic cell death and to up-regulate p53 protein expression in pancreatic cancer cell lines expressing wild-type p53 in vitro (data not shown).

Thus, p53 protein overexpression detected in cells of pancreatic juice samples from patients with pancreatitis or pancreatic cancer could be the result of DNA damage or TNF-α exposure during inflammation. Wild-type p53 protein overexpression would then be expected to lead to apoptotic cell death. Consequently we asked whether apoptotic cell death might be manifested in the cytological specimens from patients with pancreatitis or pancreatic cancer. This was in fact the case in six out of six tested cytological specimens.

The detection of p53 overexpression during pancreatitis correlates with the observation that antibodies against recombinant p53 are detectable in serum samples from patients with pancreatitis (Marxsen et al, 1994). Chronic pancreatitis is one of the rare examples in which antibodies to p53 are detectable during inflammation and in which p53 overexpression is observed.

Recently, Tada et al (1996) showed that Ki-RAS mutations occur in hyperplastic foci of the pancreatic duct, even when no pancreatic cancer or pancreatitis was demonstrable. Thus, mutation of Ki-RAS on its own cannot lead to pancreatic cancer – other genetic alterations have to occur.

Inflammatory processes during chronic pancreatitis can be expected to damage DNA. As our results suggest, this may result in p53 protein overexpression and apoptotic cell death. In some patients, a p53 mutation may occur during inflammation,

© Cancer Research Campaign 1997 

reflecting another step in the carcinogenesis of pancreatic cancer. It will be of interest to establish whether patients with a history of pancreatic carcinoma and pancreatitis have a higher degree of p53 mutation than patients without a history of pancreatitis.

### ACKNOWLEDGEMENTS

This work was supported by the E und G Roggenbuck Stiftung, Hamburg, Germany and BMBF grant (KBF)-01 GB 9502.

### REFERENCES

Almoguera C, Shibata D, Forrester K, Martin J, Arnheim N and Perucho M (1988) Most human carcinomas of the exocrine pancreas contain mutant c-K-ras genes. *Cell* **53**: 549–554

Barton CM, Staddon SL, Hughes CM, Hall PA, O'Sullivan C, Klöppel G, Theis B, Russel RCG, Neoptolemos J, Williamson RCN, Lane DP and Lemoine NR (1991) Abnormalities of the p53 tumour suppressor gene in human pancreatic cancer. *Br J Cancer* **64**: 1076–1082

Caldas C, Hahn SA, Da Costa LT, Redstone MS, Schutte M, Seymour AB, Weinstein CL, Hruban RH, Yeo CJ and Kern SE (1994) Frequent somatic mutations and homozygous deletions of the MTS1 gene in pancreatic adenocarcinoma. *Nature Genet* **8**: 27–32

Dowell SP, Wilson POG, Derias NW, Lane DP and Hall PA (1994) Clinical utility of the immunocytochemical detection of p53 protein in cytological specimens. *Cancer Res* **54**: 2914–2918

Ekbom A, McLaughlin JK, Karlson B-M, Nyrén O, Gridley G, Adami H-O and Fraumeni JF Jr (1994) Pancreatitis and pancreatic cancer: a population-based study. *J Natl Cancer Inst* **86**: 625–627

Gavrieli Y, Sherman Y and Ben-Sasson SA (1992) Identification of programmed cell death in situ via specific labelling of nuclear DNA fragmentation. *J Cell Biol* **119**: 493–501

Hahn SA, Schutte M, Hoque AT, Moskaluk CA, Da Costa LT, Rozenblum E, Weinstein CL, Fischer A, Yeo CJ, Hruban RH and Kern SE (1996) DPC4, a candidate tumour suppressor gene at human chromosome 18q21.1. *Science* **271**: 350–353

Hainaut P and Milner J (1993) Redox modulation of p53 sequence-specific DNA binding in vitro. *Cancer Res* **53**: 4469–4473

Hall PA, McKee PH, Menage HD, Dover R and Lane DP (1993) High levels of p53 protein in UV-irradiated normal human skin. *Oncogene* **8**: 203–207

Kalthoff H, Schmiegel W, Roeder C, Kasche D, Schmidt A, Lauer G, Thiele H-G, Honold G, Pantel K, Riethmüller G, Scherer E, Maurer J, Maacke H and Deppert W (1993) p53 and k-ras alterations in pancreatic epithelial cell lesions. *Oncogene* **8**: 289–298

Lane DP (1992) Cancer. p53, guardian of the genome? *Nature* **358**: 15–16

Lowenfels AB, Maisonneuve PM, Cavallini G, Ammann RW, Lankisch PG, Andersen JR, Dimagno EP, Andrén-Sandberg A and Domellöf L (1993) Pancreatitis and the risk of pancreatic cancer. *N Engl J Med* **328**: 1433–1437

Marxen J, Schmiegel W, Röder C, Harder R, Juhl H, Henne-Bruns D, Kremer B and Kalthoff H (1994) Detection of anti-p53 antibody response in malignant and benign pancreatic disease. *Br J Cancer* **70**: 1031–1034

Naumann M, Savitskaia N, Eilert C, Schramm A, Kalthoff H and Schmiegel W (1996) Frequent codeletion of p16/MTS1 and p15/MTS2 and genetic alterations in p16/MTS1 in pancreatic tumors. *Gastroenterology* **110**: 1215–1224

Schmiegel W, Burchert M, Kalthoff H, Roeder C, Bützow G, Grimm H, Kremer B, Soehendra N, Schreiber H-W, Thiele H-G and Greten H (1990) Immunochemical characterization and quantitative distribution of pancreatic stone protein in sera and pancreatic secretions in pancreatic disorders. *Gastroenterology* **99**: 1421–1430

Shibata D, Almoguera C, Forrester K, Dunitz J, Martin SE, Cosgrove MM, Perucho M and Arnheim N (1990) Detection of c-K-ras mutations in fine needle aspirates from human pancreatic adenocarcinomas. *Cancer Res* **50**: 1279–1283

Tada M, Ohashi M, Shiratori Y, Komatsu Y, Yoshida H, Machinami R, Kishi K and Omata M (1996) Analysis of K-ras gene mutation in hyperplastic duct cells of the pancreas without pancreatic disease. *Gastroenterology* **110**: 227-231

*© Cancer Research Campaign 1997*